# Comparative Efficacy and Immunogenicity of Infliximab and Adalimumab in Crohn’s Disease: A Prospective Cohort Study

**DOI:** 10.3390/medicina61122165

**Published:** 2025-12-04

**Authors:** Luis G. Guijarro, Patricia de Castro-Martínez, María Chaparro, Julio Acero-Sanz, Diego de León, Iván Guerra, Marisa Iborra, José Luis Cabriada, Luis Bujanda, Cristina Alba, Valle García-Sánchez, Ignacio Marín-Jiménez, Manuel Barreiro-de Acosta, Isabel Vera, María Dolores Martín-Arranz, Francisco Mesonero, Laura Sempere, Fernando Gomollón, Joaquín Hinojosa, Borja Hernández-Breijo, Melchor Alvarez-Mon, Javier P. Gisbert, Miguel A. Ortega

**Affiliations:** 1 Department of System Biology, University of Alcalá, 28805 Alcalá de Henares, Spain; borjahdezbreijo@gmail.com; 2 Centro de Investigación Biomédica en Red de Enfermedades Hepáticas y Digestivas (CIBEREHD), 28029 Madrid, Spain; mariachs2005@gmail.com (M.C.); javier.p.gisbert@gmail.com (J.P.G.); 3 Ramón y Cajal Institute of Sanitary Research (IRYCIS), 28034 Madrid, Spain; patriciadecastro1999@gmail.com (P.d.C.-M.); julio.acero@uah.es (J.A.-S.); mademons@gmail.com (M.A.-M.); miguel.angel.ortega92@gmail.com (M.A.O.); 4 Department of Medicine and Medical Specialties, Faculty of Medicine and Health Sciences, University of Alcalá, 28805 Alcalá de Henares, Spain; diego.leon@uah.es; 5 Gastroenterology Unit, Hospital Universitario de La Princesa, Instituto de Investigación Sanitaria Princesa (IIS-Princesa), Universidad Autónoma de Madrid (UAM), 28006 Madrid, Spain; 6 Department of Oral and Maxillofacial Surgery, Hospital Universitario Ramón y Cajal, 28034 Madrid, Spain; 7 Gastroenterology Department, Hospital Universitario de Fuenlabrada, 28942 Fuenlabrada, Spain; 8 Gastroenterology Unit, Hospital Universitario de La Fe, 46026 Valencia, Spain; marisaiborra@hotmail.com; 9 Gastroenterology Unit, Hospital Universitario de Galdakano, 48960 Galdakano, Spain; jcabriada@gmail.com; 10 Gastroenterology Unit, Hospital Universitario de Donostia, 20014 Donostia, Spain; luis.bujandafernandezdepierola@osakidetza.eus; 11 Gastroenterology Unit, Hospital Universitario Clínico San Carlos and IdISSC, 28040 Madrid, Spain; cristina.alba@salud.madrid.org; 12 Gastroenterology Unit, Hospital Universitario Reina Sofía/Universidad de Córdoba, 14004 Córdoba, Spain; vallegarciasanchez@gmail.com; 13 Gastroenterology Unit, Hospital Universitario Gregorio Marañón e IiSGM, 28007 Madrid, Spain; drnachomarin@hotmail.com; 14 Gastroenterology Unit, Hospital Universitario Clínico de Santiago, 15706 Santiago de Compostela, Spain; manubarreiro@hotmail.com; 15 Gastroenterology Unit, Hospital Universitario Puerta de Hierro Majadahonda, 28222 Majadahonda, Spain; isabel.veramendoza@gmail.com; 16 Gastroenterology Unit, Hospital Universitario La Paz, Instituto de Investigación Hospital Universitario La Paz (IdiPaz), Universidad Autónoma de Madrid, 28046 Madrid, Spain; mmartinarranz@salud.madrid.org; 17 Gastroenterology Unit, Hospital Universitario Ramón y Cajal, 28034 Madrid, Spain; pacomeso@hotmail.com; 18 Gastroenterology Unit, Hospital Universitario Alicante, 03010 Alicante, Spain; lausemro@hotmail.com; 19 Gastroenterology Unit, Hospital Clínico Universitario, Lozano Blesa, IIS Aragón, 50009 Zaragoza, Spain; fgomollon@gmail.com; 20 Gastroenterology Unit, Hospital Vithas Virgen del Consuelo, 46007 Valencia, Spain; jhinojosad@gmail.com

**Keywords:** Crohn’s disease, infliximab, adalimumab, C-reactive protein, fibrinogen, immunogenicity

## Abstract

*Background and Objectives*: Crohn’s Disease (CD) is a chronic inflammatory condition often treated with anti-TNF agents such as infliximab (IFX) and adalimumab (ADA). This study compares the efficacy, immunogenicity, and pharmacokinetics of IFX and ADA over a 54-week period. *Materials and Methods*: A prospective, multicentre cohort study was conducted involving 72 patients with active CD (Crohn’s disease activity index, CDAI > 150), who received treatment with either IFX (*n* = 42) or ADA (*n* = 30). *Results*: By week 54, treatment discontinuation occurred in 31% of IFX-treated patients (13/42) and 37% of ADA-treated patients (11/30), with no significant difference between groups (*p* = 0.612). Among those who completed the study, clinical remission (CDAI ≤ 150) was achieved in 65% of the IFX group and 95% of the ADA group (OR = 8.10; 95% CI = 1.10–20.11; *p* = 0.049). Loss of clinical response was more frequent in the IFX group (31%) than in the ADA group (10%), with an OR of 0.25 (95% CI: 0.06–0.97; *p* = 0.045). Fibrinogen levels declined in both groups, with a greater reduction observed in ADA-treated patients. The area under the ROC curve (AUC) for fibrinogen in distinguishing remission from active disease was 0.608 for IFX and 0.711 for ADA. Anti-drug antibodies were detected more frequently in IFX-treated patients (16.7%, 7/42) compared to those receiving ADA (6.7%, 2/30). *Conclusions*: Treatment with ADA demonstrated superior efficacy compared to IFX in maintaining clinical remission in CD, which was paralleled by a more effective normalization of fibrinogen levels (Clinical trial: GET-CRO-2010-01).

## 1. Introduction

Crohn’s Disease (CD) is a chronic inflammatory condition of the gastrointestinal tract that can affect any segment from the oral cavity to the anus, though it most commonly involves the small intestine and colon. It is characterized by recurrent episodes of inflammation, leading to symptoms such as abdominal pain, diarrhoea, fatigue, weight loss, and malnutrition [1].

Geographically, the incidence of CD has stabilized in many Western countries—including North America, Europe, and Australia—since the early 21st century. In contrast, a marked increase in new cases has been observed in industrializing regions, particularly across Asia and Latin America [2]. In Spain, recent estimates report a prevalence of 225 cases per 100,000 inhabitants and an incidence of 7.5 cases per 100,000 person-years [3].

The pathophysiology of CD is multifactorial, involving a complex interplay of genetic predisposition, immune dysregulation, and environmental triggers that culminate in an inappropriate immune response [4,5,6]. The advent of biologic therapies—especially anti-TNF (Tumor Necrosis Factor) agents—has significantly advanced the management of CD by targeting key inflammatory pathways [7,8]. Agents such as IFX and ADA have demonstrated high efficacy in inducing and maintaining remission in patients with CD [9,10].

Despite their effectiveness, long-term treatment with anti-TNF agents is often limited by primary non-response, secondary loss of response, or adverse effects [11]. A major contributor to these challenges is immunogenicity, wherein the formation of anti-drug antibodies reduces serum drug levels and diminishes therapeutic efficacy [12,13,14].

To optimize treatment strategies, prospective, double-blind, double-dummy clinical trials directly comparing the two biologic agents are warranted. Currently, most comparative evidence stems from retrospective studies [15] and a limited number of prospective observational cohort studies [16], which do not find significant differences in the efficacy or safety profiles of IFX and ADA.

This article presents a clinical study designed to compare the efficacy, safety, and immunogenicity of IFX and ADA in the treatment of CD. The study assesses drug trough levels, anti-drug antibody formation, and TNFα concentrations, and explores their correlation with clinical outcomes, including the clinical activity and relevant haematological and biochemical markers, among which are acute-phase proteins such as fibrinogen and C-reactive protein (CRP), given their close link to systemic and vascular inflammation in Crohn’s disease. A deeper understanding of these parameters is crucial for tailoring individualized therapeutic strategies and enhancing long-term disease management.

## 2. Patients and Methods

### 2.1. Study Design and Setting

This prospective, multicentre cohort study included 150 consecutive patients diagnosed with CD in accordance with the diagnostic criteria established by the European Crohn’s and Colitis Organisation (ECCO) [17]. All participants were initiating anti-TNF therapy for clinical indications as part of the PREDICROHN study (GET-CRO-2010-01), approved by the research division of the Grupo Español de Tra-bajo en Enfermedad de Crohn y Colitis Ulcerosa (GETECCU) and by the Clinical Research Ethics Committee of Hospital de la Princesa (Approval Code: GET-CRO-2010-01, Approval Date: 23 September 2010). The primary objective of the Predicrohn study was to optimize therapeutic response to anti-TNF agents by identifying biomarkers of clinical efficacy, including serum drug levels, anti-drug antibodies, and TNFα concentrations.

Inclusion and exclusion criteria: At baseline, patients were eligible for inclusion if they met the following criteria: (a) Age ≥ 18 years; (b) Confirmed diagnosis of CD based on clinical, radiological, endoscopic, and/or histological findings; (c) Indication for anti-TNF therapy; (d) Written informed consent; (e) Crohn’s Disease Activity Index (CDAI) > 150.

**Figure 1:**
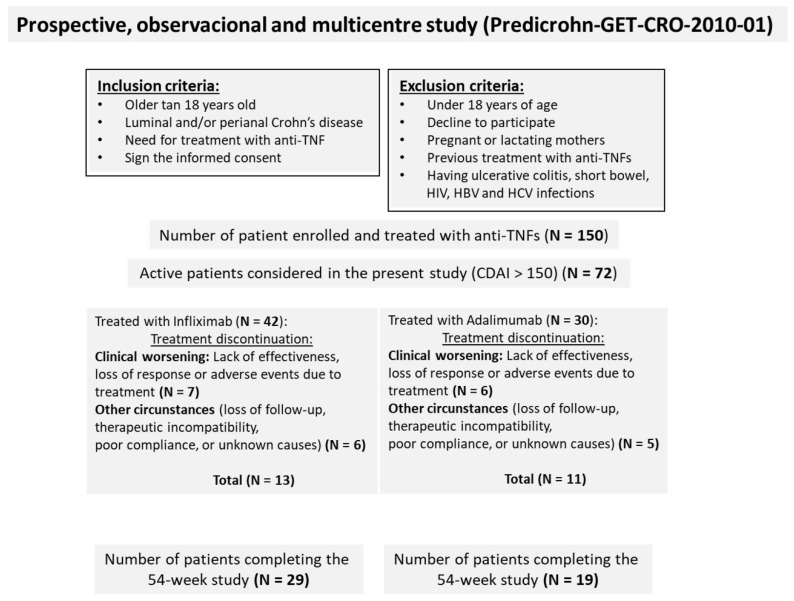
Study flow diagram illustrating patient progression from enrolment to final analysis.

Exclusion criteria included: (a) Pregnancy or breastfeeding; (b) Prior biologic therapy; (c) Diagnosis of ulcerative colitis; (d) Short bowel syndrome; (e) Contraindications to biologic treatment; (f) Presence of an ostomy; (g) Abdominal surgery within the past 6 months; (h) Active hepatitis B or C infection; (i) HIV infection; (j) Positive tuberculin skin test; (k) Anti-TNF therapy indicated for conditions other than CD. Treatment allocation (IFX or ADA) was determined by the treating physician and the patient through a shared clinical decision-making process, reflecting real-world clinical practice, and was not randomized. From the initial cohort of 150 patients, a nested study cohort of 72 patients with CDAI > 150 at baseline was selected for the present study (Figure 1). Only patients with CDAI >150 were included in this analysis because this threshold is widely accepted to define active Crohn’s disease, and the primary objective was to compare the ability of IFX and ADA to induce remission in active patients.

Treatment Protocol:

IFX Group: patients received 5 mg/kg intravenously at weeks 0 (visit 1 or baseline), 4 (visit 2), 8 (visit 3), 14 (visit 4), 22 (visit 5), 30 (visit 6), 38 (visit 7), 46 (visit 8) and 54 (visit 9).

ADA Group: patients received 160 mg subcutaneously at week 0 (visit 1 or baseline), 80 mg at week 2 (visit 2), and 40 mg at weeks 6 (visit 3), and every 8 weeks thereafter: weeks 14 (visit 4), 22 (visit 5), 30 (visit 6), 38 (visit 7), 46 (visit 8) and 54 (visit 9).

Clinical and laboratory assessments:

At each scheduled visit, prior to drug administration, patients underwent clinical evaluation and blood sampling. The following parameters were assessed:

Biochemical markers (serum): albumin, ferritin, C-reactive protein (CRP), and fibrinogen.

Haematological markers (whole blood): haemoglobin, leukocyte count, neutrophils, platelets, and erythrocyte sedimentation rate (ESR).

All routine laboratory analyses were conducted at the clinical laboratories of the participating hospitals. Additionally, serum samples were collected for centralized measurement of TNFα, IFX, ADA, and anti-drug antibodies (anti-infliximab antibodies [ATI] and anti-adalimumab antibodies [ATA]). These immunological assays were performed by Prometheus Laboratories Inc. (San Diego, CA, USA, https://prometheuslabs.com/) following previously validated protocols. The limit of quantification (LOQ) was 0.98 µg/mL for IFX and 3.13 U/mL for ATI. For ADA, the LOQ was 1.0 µg/mL, and for ATA, it was 3.13 U/mL. Values less than or equal to the LOQ were treated as zero.

### 2.2. Outcomes and Definitions

The primary clinical outcome of this study was the proportion of patients achieving clinical remission, defined as a Crohn’s Disease Activity Index (CDAI) ≤ 150.

Secondary effectiveness outcomes included treatment discontinuation rate, incidence of adverse events, loss of clinical response—defined as a CDAI increase of ≥100 points from the lowest recorded value (clinical recurrence)—and stabilization or improvement of haematological and biochemical parameters, including haemoglobin, leukocyte count, CRP, albumin, among others.

The second primary outcome was the evaluation of immunogenicity of both anti-TNF agents. This was assessed through: Detection of anti-drug antibodies: ATI and ATA. Additional pharmacokinetic outcomes included: Serum concentrations of IFX and ADA, serum levels of TNFα, the therapeutic target of both agents. These outcomes were measured at predefined time points throughout the 54-week follow-up period, allowing for longitudinal assessment of treatment efficacy, safety, and immunogenicity.

### 2.3. Statistical Analysis

All statistical analyses were conducted using R software (R Project for Statistical Computing, version 4.3.3; https://www.R-project.org).

To evaluate baseline comparability between the IFX and ADA groups, the Mann–Whitney U test was applied for continuous variables, and the chi-square test for categorical variables.

A multiple logistic regression model (LOG model) was used to assess the primary effectiveness outcome—clinical remission—while adjusting for potential confounding variables. Candidate covariates included: treatment discontinuation, baseline CDAI, history of intestinal surgery, presence of extraintestinal manifestations, disease behaviour, concomitant corticosteroid use, smoking status.

Each variable was individually introduced into the model to assess its influence on the dependent variable (CDAI). Although several candidate covariates were evaluated, none except treatment discontinuation altered the odds ratio by ≥15%, suggesting limited association with the primary outcome; therefore, only treatment discontinuation was retained in the final model. To analyse longitudinal changes in haematological and biochemical parameters between treatment groups, a mixed linear regression model (LIN model) was employed. The mixed-effects model assumed normal distribution for continuous outcomes and included patient-level random intercepts to account for correlation among repeated measures. The same modelling approach was applied to assess temporal variations in: serum drug concentrations (IFX and ADA), anti-drug antibody levels (ATI and ATA), TNFα serum levels.

The relationship between biochemical markers and disease activity was assessed by comparing their levels in patients with active Crohn’s disease (CD) (CDAI > 150) and those in remission (CDAI ≤ 150). Only parameters that showed significant differences between patients treated with IFX and ADA were considered.

Patients receiving each treatment were categorized at each visit based on disease activity status—either in remission or with active disease. Subsequently, levels of the therapeutic drugs, anti-drug antibodies, TNFα, and fibrinogen were compared between these groups.

To explore the relationship between serum biomarkers (drug levels, anti-drug antibodies, and TNFα) and disease activity status (active disease vs. remission), the Mann–Whitney U test was used. Statistical significance was defined as follows: *p* < 0.05 (*).

### 2.4. Ethical Considerations

This study was conducted in full compliance with the ethical principles outlined in the Declaration of Helsinki (1975) and its subsequent amendments. Ethical approval was obtained from the Institutional Ethics Committees of all participating hospitals prior to the initiation of the study. All participants provided written informed consent through a research authorization form at the time of their clinical evaluation, ensuring voluntary participation and understanding of the study procedures and objectives. The study protocol was formally approved and authorized by the Grupo Español de Trabajo en Enfermedad de Crohn y Colitis Ulcerosa (GETECCU), under the Predicrohn initiative (https://geteccu.org/predicrohn).

Generative AI tools (specifically Microsoft Copilot) were used to assist in the drafting and language refinement of this manuscript. All scientific content, interpretations, and conclusions were reviewed and validated by the authors.

## 3. Results

### 3.1. Baseline Characteristics

A total of 150 patients were enrolled in the PREDICROHN clinical study, with 78 receiving IFX and 72 receiving ADA. Of these, 72 patients met the inclusion criteria for this study, defined as having a Crohn’s Disease Activity Index (CDAI) greater than 150. Among them, 42 patients were treated with IFX and 30 with ADA (Figure 1). The baseline characteristics of patients treated with IFX and ADA are summarized in Table 1.

No significant differences were observed between the two groups in terms of age, sex, or smoking status. Although a statistically significant difference in weight was noted, both height and body mass index (BMI) were comparable across groups. Clinical disease activity parameters, including the CDAI and the HBI, did not differ significantly between groups. Similarly, no significant differences were found in disease behaviour, the prevalence of extraintestinal manifestations, or the proportion of patients with prior surgical interventions. Finally, haematological and biochemical parameters were comparable between the two treatment groups, with no statistically significant differences observed.

### 3.2. Efficacy, Treatment Discontinuation and Clinical Remission

**Figure 2:**
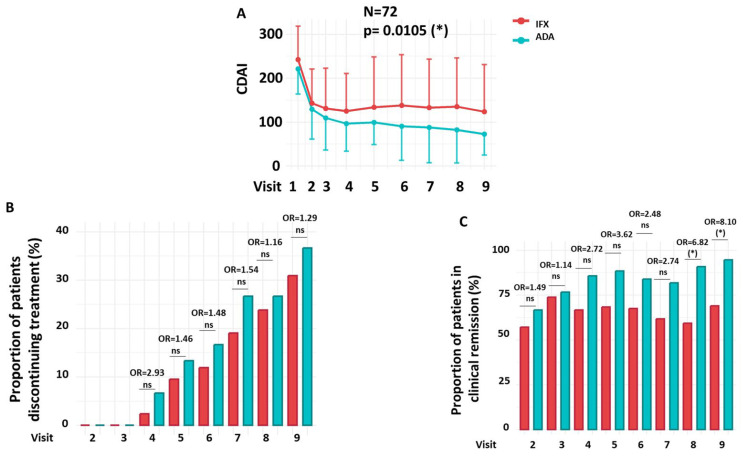
Efficacy, Treatment Discontinuation, and Clinical Remission. (**A**) CDAI progression throughout the clinical trial. (**B**) Proportion of patients who discontinued treatment. (**C**) Proportion of patients achieving clinical remission (CDAI ≤ 150) over the course of treatment. Longitudinal changes between treatment groups observed in panel A were analyzed using a mixed-effects linear regression model (LIN model), while changes in panels (**B**,**C**) were assessed using a multiple logistic regression model (LOG model). Details of both LIN and LOG models are provided in the Patients and Methods section. Odds ratios (OR) at each visit and the overall OR were estimated from the LOG model; the overall OR reflects the cumulative effect across all visits after adjusting for treatment discontinuation. Red = IFX; Green = ADA. OR = Odds Ratio. N = Number of patients. ns = not significant. * *p* < 0.05.

This section evaluates the therapeutic efficacy of IFX and ADA over a 54-week period, focusing on the proportion of patients achieving clinical remission and the rates of treatment discontinuation. A reduction in CDAI scores was observed in CD patients treated with either IFX or ADA over the 54-week period. However, the extent of the reduction was significantly greater in patients treated with ADA than in those receiving IFX (Figure 2A).

During the 54-week follow-up period, there were no statistically significant differences in the proportion of patients who discontinued treatment between the IFX and ADA groups (Figure 2B). At the final study visit (Week 54), treatment discontinuation was observed in 31.0% of patients receiving IFX (*n* = 13/42) and 36.7% of those receiving ADA (*n* = 11/30). Throughout the follow-up period, no statistically significant differences in discontinuation rates were observed between the two groups at any time point (OR = 1.05; 95% CI: 0.80–1.38; *p* = 0.732).

Clinical remission rates, defined as a CDAI ≤ 150, were significantly different between the IFX and ADA groups throughout the study (OR = 1.31; 95% CI: 1.11–1.55; *p* = 0.002). As shown in Figure 2C, statistically significant differences in remission were observed at Visit 8 (59.38% vs. 90.91%; OR = 6.82; 95% CI = 1.45–25.39; *p* = 0.019) and Visit 9 (65.52% vs. 94.74%; OR = 8.10; 95% CI = 1.10–20.11; *p* = 0.049), favouring the ADA-treated group.

### 3.3. Adverse Events, Loss of Clinical Response, and Reasons for Discontinuation

No statistically significant difference was observed in the overall incidence of adverse events between the IFX and ADA groups (OR = 0.40; 95% CI: 0.13–1.27; *p* = 0.119) (Table 2). In the IFX group, 14 out of 42 patients (33%) experienced adverse events, including infections (*n* = 5), skin lesions (*n* = 3), pain (*n* = 3), joint disease (*n* = 2), and digestive disorders (*n* = 1). In comparison, 5 out of 30 patients (17%) in the ADA group reported adverse events, primarily skin lesions (*n* = 2), joint disease (*n* = 1), infections (*n* = 1), and pain (*n* = 1). A significant difference was observed in the loss of clinical response, with 31% (13/42) of IFX-treated patients affected compared to 10% (3/30) in the ADA group (OR = 0.25; 95% CI: 0.06–0.97; *p* = 0.045), favouring ADA.

Regarding treatment discontinuation, no significant difference was found between the two groups (OR = 1.29; 95% CI: 0.48–3.47; *p* = 0.612). The most common reason for discontinuation in both groups was clinical worsening (6/13 in IFX vs. 6/11 in ADA). Other reasons included loss to follow-up, poor compliance, or unrelated causes (7/13 vs. 5/11, respectively) (Table 2).

### 3.4. Haematological and Biochemical Parameters

**Figure 3:**
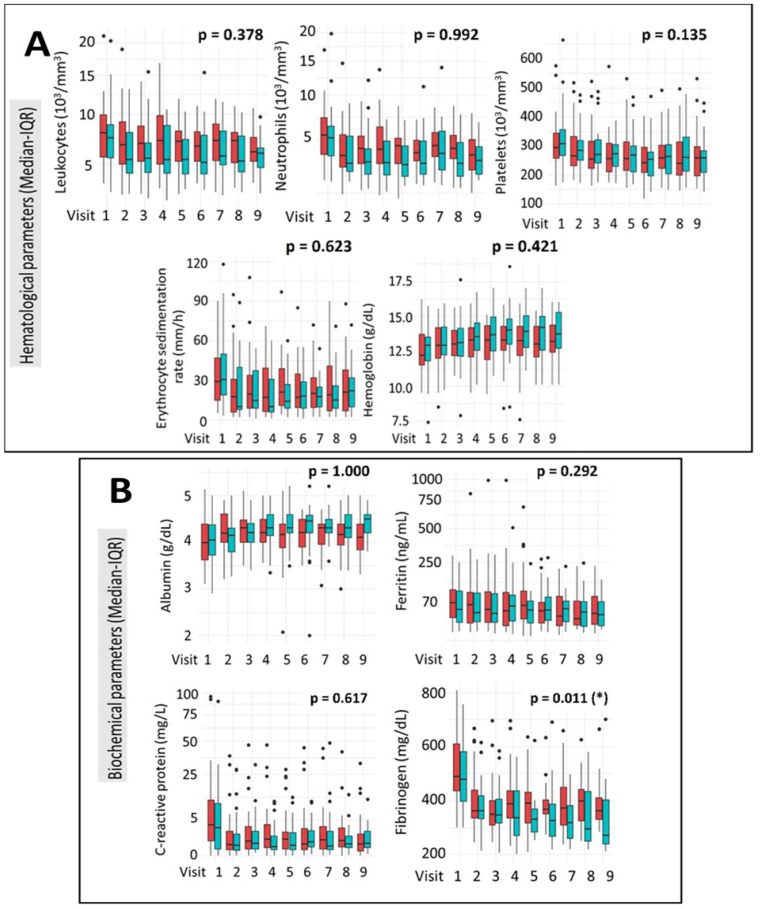
Evolution of Haematological (**A**) and Biochemical (**B**) Parameters During the Clinical Trial. Values are presented as median (interquartile range, IQR). Longitudinal changes between treatment groups were assessed using a mixed-effects linear regression model (LIN model), as detailed in Section 2. IFX = red; ADA = green. * *p* < 0.05.

Comparative analysis of haematological profiles showed no statistically significant differences between the IFX and ADA treatment groups throughout the study period. Parameters assessed included leukocyte and neutrophil counts, platelet levels, erythrocyte sedimentation rate (ESR), and haemoglobin concentration (Figure 3A). Analysis of biochemical parameters revealed no statistically significant differences between the IFX and ADA groups in albumin, ferritin, or C-reactive protein (CRP) levels throughout the study. CRP levels demonstrated a significant downward trend throughout the clinical study in both treatment groups. However, a statistically significant difference in fibrinogen levels was observed between the groups over the course of the study, with a more pronounced reduction in the ADA group compared to the IFX group (*p* = 0.011) (Figure 3B).

### 3.5. Pharmacological Monitoring, Immunogenicity and TNFα Levels

**Figure 4:**
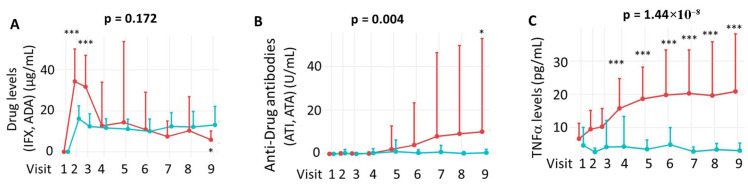
Pharmacological monitoring (**A**), immunogenicity (**B**) and TNFα (**C**) levels throughout the clinical trial. Panel (**A**) shows drug concentration levels over time; statistical comparisons at each visit were performed using the Mann–Whitney U test. Panel (**B**) illustrates the development of anti-drug antibodies, and panel (**C**) displays TNFα levels during the study period. Longitudinal changes between treatment groups were assessed using a mixed-effects linear regression model (LIN model), as detailed in the Patients and Methods section. IFX = Infliximab. ADA = Adalimumab. ATI = anti-infliximab antibodies. ATA = anti-adalimumab antibodies. The comparison between visits was performed using the Mann–Whitney U test. IFX treatment = red; ADA treatment = green. * *p* < 0.05; *** *p* < 0.001.

Pharmacological monitoring of anti-TNF agents is presented in Figure 4A, showing serum drug concentrations over time. The immunogenic response, measured by the presence of anti-drug antibodies, is depicted in Figure 4B, while Figure 4C illustrates serum levels of the therapeutic target, TNFα.

Using a mixed linear regression model (LIN model), no significant differences were found in overall serum concentrations of IFX and ADA across the study period (*p* = 0.172). As shown in Figure 4A, ADA levels remained stable within a narrow range, whereas IFX levels fluctuated depending on the timing of administration.

To further explore these dynamics, drug levels were compared at individual visits. As illustrated in Figure 4A, IFX levels were significantly higher than ADA levels at Visits 2 and 3 (*p* < 0.001), while ADA levels were higher at Visit 9 (*p* < 0.05). Regarding immunogenicity, the incidence of anti-drug antibodies was significantly higher for ATI than for ATA throughout the study (*p* = 0.004), with ATI detected in 7 of 42 IFX-treated patients and ATA in 2 of 30 ADA-treated patients. At the 54th week of treatment, a significantly higher incidence of ATI was observed compared to ATA (*p* < 0.05) when analysing individual visits, highlighting increased immunogenicity in the IFX group (Figure 4B). Finally, TNFα levels were significantly higher in the IFX group compared to the ADA group throughout the clinical study (*p* = 1.44 × 10^−8^), which likely reflects detection of TNFα–infliximab complexes by the ELISA method rather than active cytokine. This difference became statistically significant starting at Visit 4 (week 14) of treatment (Figure 4C).

### 3.6. Correlation of Biomarkers with Disease Activity

**Figure 5:**
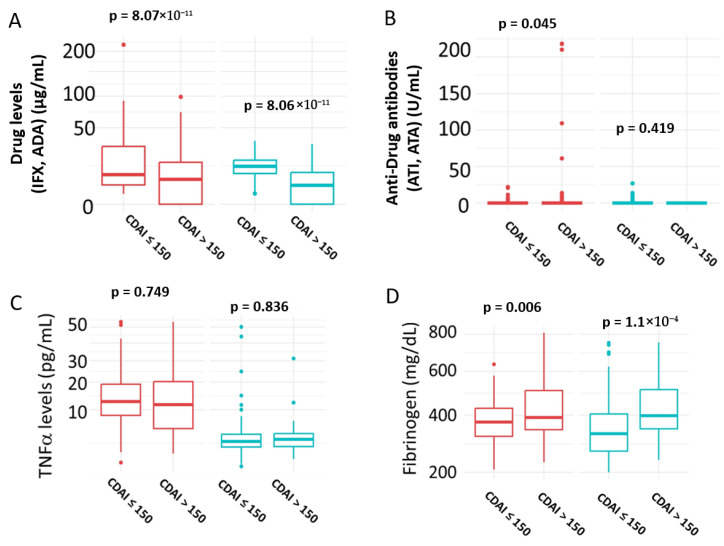
Correlation of Biomarkers with Disease Activity. (**A**) Serum Drug levels; (**B**) Serum anti-Drug antibodies levels; (**C**) Serum TNFα levels; (**D**) serum fibrinogen levels. Patients in remission were defined as those with CDAI ≤ 150; active disease was defined as CDAI > 150. IFX = Infliximab; ADA = Adalimumab; ATI = anti-infliximab antibodies; ATA = anti-adalimumab antibodies. IFX treatment = red; ADA treatment = green. Statistical significance was assessed using the Mann–Whitney U test.

The results are presented in Figure 5. Patients in remission exhibited significantly higher serum drug levels in both the IFX (*p* = 8.07 × 10^−11^) and ADA (*p* = 8.06 × 10^−11^) groups (Figure 5A). In contrast, anti-drug antibody levels were significantly elevated in patients with active disease compared to those in remission in the IFX group (*p* = 0.045), but not in the ADA group (*p* = 0.419) (Figure 5B). Serum TNFα levels did not show a significant association with disease activity in either treatment group (IFX: *p* = 0.749; ADA: *p* = 0.836) (Figure 5C). Serum TNFα did not show a significant association with disease activity (AUC not significant), supporting that measured TNFα represents complexed rather than biologically active cytokine. However, fibrinogen levels were positively correlated with disease activity, with significantly higher levels observed in patients with active disease in both the IFX (*p* = 0.006) and ADA (*p* = 1.1 × 10^−4^) groups (Figure 5D).

**Figure 6:**
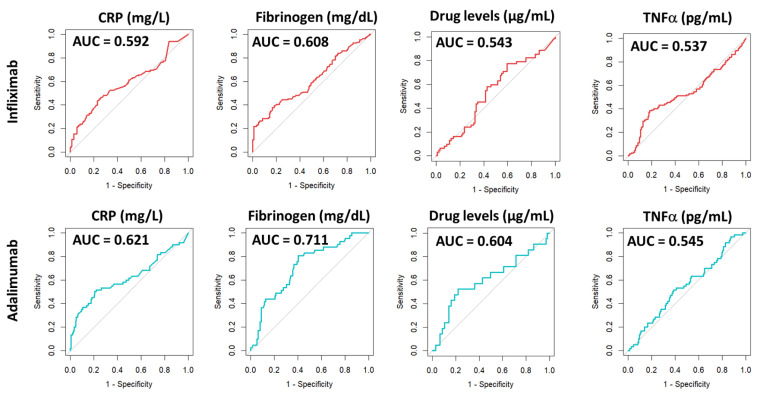
ROC curve analysis of biomarkers associated with patients in remission (CDAI ≤ 150) or with active disease (CDAI > 150). AUC = area under curve. CRP = C-reactive protein.

We then assessed the discriminatory power of the selected parameters to differentiate between active and remission states in Crohn’s disease patients. To this end, ROC curve analyses were performed for drug levels, anti-drug antibodies, the therapeutic target (TNFα), as well as fibrinogen and C-reactive protein levels, which served as reference controls. The results are depicted in Figure 6.

As shown in Table 3, the evaluation of the AUC for drug levels, anti-drug antibodies, and TNFα did not reach statistical significance for either IFX or ADA, suggesting that these markers are not effective in distinguishing between active disease and remission. However, when analysing the AUC for CRP and fibrinogen levels, statistically significant values were obtained, indicating a significant discriminatory ability regarding the patient’s Crohn’s disease activity in both IFX and ADA groups.

## 4. Discussion

In this prospective, real-world study, ADA achieved higher remission rates than IFX in CD patients over 54 weeks. This difference may be partially explained by ADA’s greater capacity to normalize fibrinogen levels. Several recent systematic reviews and meta-analyses report comparable efficacy between the two drugs, both in the treatment of perianal CD [18], in the prevention of postoperative recurrence [19] and in overall disease activity [20].

However, these conclusions are drawn from heterogeneous clinical trials with varying designs, which limits the possibility of direct comparisons. Additional evidence comes from retrospective cohort studies based on medical records [15,21], which often include a mix of anti-TNF-naïve and previously treated patients [16]. There are very few prospective studies specifically designed to examine the differences between IFX and ADA in both the short and long term. To address these limitations, our study exclusively included patients with no prior anti-TNF treatment, as confirmed by baseline drug level testing. However, the non-randomized allocation of treatment, determined by the physician and patients through a shared clinical decision-making process rather than by random assignment, represents a potential source of allocation bias. Although efforts were made to balance recruitment across participating physicians, this limitation should be considered when interpreting the comparative efficacy results. Additionally, the sample size (*n* = 72), while appropriate for a pilot comparative study, limits statistical power for certain secondary outcomes such as ATA/ATI incidence. Future studies should incorporate formal power calculations and effect size estimates to better contextualize these findings and strengthen the interpretation of secondary endpoints.

The study duration was longer than that of a previous prospective open-label studies comparing ADA and IFX [9], which enhances the ability to compare the remission rates and the safety profile of both drugs. Although our findings suggest that ADA may be more effective in maintaining clinical remission, confirmation through larger, double-blind, double-dummy trials with extended follow-up periods is needed to validate these results and support their application in routine clinical practice. The overall odds ratio (OR = 1.31; 95% CI: 1.11–1.55), obtained from the longitudinal model adjusting for treatment discontinuation, differs from the unadjusted visit-specific ORs at Visit 8 (OR = 6.82; 95% CI: 1.45–25.39) and Visit 9 (OR = 8.10; 95% CI: 1.10–20.11). This difference reflects that the global estimate summarizes the cumulative effect across all visits and accounts for treatment discontinuation, whereas raw visit-level ORs capture isolated time points. When a treatment shows a consistent advantage over time, the longitudinal model amplifies this effect by integrating repeated measures and reducing variability due to discontinuation.

Discontinuation and adverse event frequencies were comparable between IFX and ADA at 54 weeks, aligning with previous studies that report similar safety profiles [14,22,23]. However, the rate of loss of clinical response differed significantly, favouring ADA over IFX. This observation is consistent with earlier findings [24,25,26]. The secondary loss of response to IFX is frequently associated with the formation of anti-drug antibodies (ATIs), a phenomenon attributed to its relatively higher immunogenicity compared to ADA. Recent reports suggest that the underlying causes may be associated with genetic factors such as the HLA-DQA1*05 allele [27], the FCGR3A polymorphism [28], or the HLA-DRB1 allele [29].

In our study, we observed a higher proportion of ATIs compared to ADAs. However, the predictive value of anti-drug antibody levels could not be confirmed through ROC analysis, likely due to the limited number of patients who developed immunogenicity. We observed a positive correlation between clinical remission and higher drug trough levels for both treatments. However, IFX concentrations were significantly lower than those of ADA at week 54, coinciding with peak levels of ATIs. As previously reported, IFX and ATI levels exhibit an inverse relationship in patients with Crohn’s disease, whereby higher ATI concentrations are associated with lower circulating levels of IFX [30]. However, the ability of serum IFX or ADA levels to reliably discriminate between clinical remission and active disease using receiver operating characteristic (ROC) curve analysis did not reach statistical significance.

Among all haematological and biochemical parameters evaluated, fibrinogen appeared to most consistently reflect the differences between IFX and ADA over the 54-week clinical study. Both IFX and ADA significantly reduced fibrinogen levels throughout the study, although ADA showed a greater effect. This observation aligns with previous findings identifying fibrinogen as a potential biomarker of active Crohn’s disease [31]. Anti-TNF therapy may contribute to the restoration of coagulation and haemodynamic balance, in part by normalising fibrinogen levels and platelet aggregation. These effects are likely mediated by TNFα blockade, which reduces local microvascular resistance and enhances blood flow in the superior mesenteric artery (SMA)—a vessel that supplies a substantial portion of the intestines and can be compromised by inflammation observed in Crohn’s disease (CD). These vascular improvements seem to progress concurrently with the decrease in CDAI [32]. Fibrinogen reflects systemic and local vascular inflammation, processes closely linked to disease activity and thrombotic risk in Crohn’s disease. In addition, fibrinogen showed a fair discriminatory ability for clinical remission (AUC = 0.711), slightly higher than CRP (AUC = 0.621), suggesting that it may serve as a complementary marker of therapeutic response rather than a definitive explanation for ADA’s superior efficacy.

In our study, baseline serum TNFα levels were comparable across groups. Patients treated with IFX exhibited a markedly higher increase in TNFα compared to those receiving ADA, most likely due to assay detection of TNFα bound to infliximab rather than active cytokine. This analytical phenomenon explains the observed difference and does not indicate a causal role in clinical outcomes. Despite this, receiver operating characteristic (ROC) curve analysis did not demonstrate a statistically significant predictive value of serum TNFα levels for distinguishing between remission and active disease in patients with Crohn’s disease. This may be due to the fact that, in Crohn’s disease, the soluble form of TNFα is not necessarily clinically relevant [33]. Instead, the therapeutic efficacy of anti-TNF agents likely relies on targeting the transmembrane form of TNFα, which appears to play a more pivotal role in modulating intestinal inflammation and inducing apoptosis in pathogenic T cells [34].

This study has several limitations. Treatment allocation was not randomised but based on physician–patient decision, which may introduce allocation bias. The nested cohort was relatively small, limiting statistical power, particularly for immunogenicity analyses. As an observational design, causal relationships cannot be established. Variability in clinical practice among participating physicians may have influenced treatment decisions and outcomes. Finally, as this was a multicentre Spanish cohort, findings may primarily reflect regional clinical practice and patient characteristics; external validation in other settings is warranted to confirm generalisability. Despite these limitations, the study provides valuable real-world evidence comparing IFX and ADA in active Crohn’s disease, complementing data from randomised trials and informing clinical decision-making.

## 5. Conclusions

This study highlights the superior efficacy of ADA over IFX in maintaining clinical remission in Crohn’s disease. Although both agents showed similar rates of treatment discontinuation due to adverse events or reduced efficacy, ADA was associated with a lower incidence of secondary loss of response. Further research is warranted to elucidate the long-term impact of immunogenicity and to enhance treatment durability. Standardization of anti-drug antibody assays remains a critical step toward this goal. Additionally, our findings support the incorporation of fibrinogen measurement into routine therapeutic drug monitoring, potentially enabling more personalized and timely adjustments in anti-TNFα therapy.

## Figures and Tables

**Table 1:** Baseline demographic and clinical characteristics of patients with active disease (CDAI > 150), categorized according to biologic therapy received.

	Infliximab (*n* = 42)	Adalimumab (*n* = 30)	*p*-Value
Age (years). Median (IQR)	33.0 (25.0–53.8)	38.5 (30.8–45.8)	0.70
Sex ✓ Men. N (%) ✓ Women. N (%)	19 (45%) 23 (55%)	18 (60%) 12 (40%)	0.31
Smoking habit. N (%)	15 (36%)	10 (33%)	1.00
Weight (kg). Median (IQR)	59.9 (53.9–64.8)	66.0 (59.0–76.4)	0.04
Height (cm). Median (IQR)	165.0 (156.0–172.0)	170.0 (163.0–176.0)	0.06
Body mass index (kg*m^−2^). Median (IQR)	22.1 (19.9–24.2)	22.7 (20.7–25.8)	0.28
Clinical parameters ✓ CDAI. Median (IQR) ✓ HBI. Median (IQR)	223.3 (187.0–283.0) 7.0 (5.0–8.8)	200.0 (173.3–267.3) 6.5 (5.0–8.0)	0.23 0.15
Behavior ✓ Inflammatory. N (%) ✓ Stricturing. N (%) ✓ Fistulizing. N (%)	28 (66%) 2 (5%) 12 (29%)	26 (87%) 0 (0%) 4 (13%)	0.12
Extraintestinal manifestations. N (%)	16 (38%)	10 (33%)	0.87
Previous surgery. N (%)	18 (43%)	10 (33%)	0.57
Hematological parameters (Median-IQR) ✓ Hemoglobin (g/dL). ✓ Leukocytes (10^3^/mm^3^). ✓ Neutrophils (10^3^/mm^3^). ✓ Platelets (10^3^/mm^3^). ✓ ESR (mm/h).	12.2 (11.5–13.7) 7.9 (5.6–9.9) 5.4 (3.7–7.3) 294.0 (257.0–345.0) 29.0 (15.3–46.8)	12.9 (11.8–4.4) 7.4 (5.5–8.8) 5.1 (3.6–6.3) 307.0 (266.3–356.8) 30.5 (19.0–50.0)	0.32 0.18 0.40 0.44 0.87
Biochemical parameters (Median-IQR) ✓ Albumin (g/dL). ✓ Ferritin (ng/mL). ✓ C-reactive protein (mg/L). ✓ Fibrinogen (mg/dL).	4.0 (3.6–4.4) 65.5 (28.0–113.4) 3.5 (0.9–11.7) 488.5 (437.3–610.5)	4.1 (3.7–4.4) 46.4 (19.9–107.9) 3.0 (0.2–10.3) 477.0 (398.5–581.0)	0.67 0.21 0.44 0.64

**Table 2:** Adverse events, loss of clinical response and reasons for discontinuation.

	Infliximab (*n* = 42)	Adalimumab (*n* = 30)	Odds Ratio
Adverse events ✓ Skin (hair loss, eczema, psoriatic lesions) ✓ Joints (arthritis, arthralgia) ✓ Infections (candidiasis, tuberculosis, fungal infection, pharyngitis, otitis) ✓ Digestive tract (pharyngeal itch) ✓ Pain (headache, precordial pain)	14/42 (33%) 3 2 5 1 3	5/30 (17%) 2 1 1 0 1	0.40 *p* = 0.119
Loss of clinical response	13/42 (31%)	3/30 (10%)	0.25 *p* = 0.045
Treatment discontinuation ✓ Clinical worsening (lack of effectiveness, loss of response or adverse events due to treatment) ✓ Other circumstances (loss of follow-up, therapeutic incompatibility or unknown causes not attributable to treatment)	13/42 (31%) 6 7	11/30 (37%) 6 5	1.29 *p* = 0.612

**Table 3:** Results from ROC analysis of biomarkers associated with patients in remission (CDAI ≤ 150) or with active disease (CDAI > 150).

Infliximab	Threshold	Youden’s Index	Auc	Specificity	Sensitivity	Statistical Level
C-reactive protein	2.5 mg/L	0.206	0.592	0.770	0.435	*p* < 0.05 (*)
Fibrinogen	442.0 mg/dL	0.213	0.608	0.817	0.396	*p* < 0.05 (*)
Drug levels	5.1 µg/mL	0.179	0.543	0.404	0.774	n.s.
TNFα	6.9 pg/mL	0.202	0.537	0.818	0.384	n.s.
**Adalimumab**	**Threshold**	**Youden’s index**	**AUC**	**Specificity**	**Sensitivity**	**Statistical level**
C-reactive protein	1.8 mg/L	0.297	0.621	0.780	0.517	*p* < 0.05 (*)
Fibrinogen	344.0 mg/dL	0.403	0.711	0.598	0.805	*p* < 0.05 (*)
Drug levels	9.5 µg/mL	0.305	0.604	0.781	0.524	n.s.
TNFα	3.1 pg/mL	0.131	0.545	0.614	0.520	n.s.

AUC = area under curve. Optimal thresholds calculated using Youden’s index for each biomarker. n.s. = not significant. * *p* < 0.05.

## Data Availability

Data are available from the corresponding author upon reasonable request and subject to institutional and ethical guidelines.
